# Analysis of the chain-mediated effects of nurses’ sense of professional gain and sense of professional mission between psychological resilience and work engagement in 10 general hospitals in Sichuan province

**DOI:** 10.3389/fpsyg.2024.1309901

**Published:** 2024-01-23

**Authors:** Zhenfan Liu, Cui Chen, Xiaoting Yan, Jijun Wu, Lin Long

**Affiliations:** ^1^Department of Nursing, Deyang People’s Hospital, Deyang, China; ^2^Department of Nursing, Sichuan Nursing Vocational College, Chengdu, China; ^3^School of Nursing, Sichuan North Medical College, Nanchong, China

**Keywords:** nurses, psychological resilience, sense of professional gain, sense of professional mission, work engagement, chain mediating effects

## Abstract

**Objective:**

To explore the chain-mediated role of sense of career benefit and sense of career mission in the mechanism of psychological flexibility’s effect on nurses’ work engagement.

**Methods:**

Adopting the convenience sampling method, 1032 nurses in 10 general hospitals in Sichuan Province were surveyed by questionnaires using the General Information Questionnaire, Sense of Occupational Benefit Scale, Sense of Occupational Mission Scale, Psychological Flexibility Scale, and work engagement Scale in August-October 2022, and the model of the chained-mediated effect was constructed and validated.

**Results:**

The total psychological resilience score of nurses in 10 general hospitals in Sichuan Province was (91.29 ± 17.38), the total score of sense of occupational benefit was (137.85 ± 21.02), the total score of sense of occupational mission was (40.27 ± 7.37), and the total score of work engagement was (34.99 ± 9.80). The total score of nurses’ work engagement was positively correlated with the total scores of psychological elasticity, sense of professional benefit, and sense of professional mission (all *P* < 0.05). The direct effect of psychological elasticity on nurses’ work engagement was significant, with an effect value of 0.321; the chain mediation effects of occupational benefit and occupational mission as separate mediators and the chain mediation effects of the two were 0.039, 0.032, and 0.062, respectively.

**Conclusion:**

Nurses’ work engagement in 10 general hospitals in Sichuan province is at a medium level, and occupational benefit and occupational mission play a significant role in the mechanism of the psychological elasticity’s effects on nurses’ work commitment, and the chain mediation effect of occupational mission in the mechanism of psychological elasticity is established. The chain mediation effect in the mechanism was established. Managers should pay attention to nurses with low psychological elasticity, improve their sense of occupational benefit, and enhance their sense of occupational mission in order to further promote the enhancement of work engagement.

## 1 Introduction

Currently, with the continuous development of Chinese economy, people’s awareness of their own health management is increasing, and nurses not only need to meet the basic medical needs of patients, but also need to provide high-quality nursing services for patients. While this brings development opportunities to nurses, it also creates a huge workload for nurses, which makes nurses under increased pressure, prone to negative burnout, and even inclined to leave their jobs (Zhou et al., 2023). As we all know, the phenomenon of high turnover rate of nurses is common all over the world, and the turnover rate of nurses around the world ranges from 15 to 44% (Pang et al., 2020; Wang D. et al., 2022). Although the base number of nurses in China is large, the manpower allocation is far from enough, and there is still a high departure rate (Liu et al., 2023). It is essential to reduce the turnover rate of nurses.

Work engagement refers to the positive emotional and cognitive state that an individual displays at work, consisting of concentration, dedication, and vigor. Currently, work engagement has been extensively researched in the field of management (Abu Dalal et al., 2022). A positive state of work engagement can effectively enhance nurses’ job satisfaction, reduce turnover rates, and stabilize the nursing workforce (Liu et al., 2022). Paying attention to nurses’ work engagement plays an important role in improving the quality of clinical care and ensuring patient safety (Parr et al., 2021).

Nurses’ work engagement is affected by a variety of factors, and psychological resilience is one of the important influencing factors. As a positive psychological resource, psychological resilience mainly refers to an individual’s adaptive ability to effectively face and adapt to the dilemmas and pressures brought about by work or life (Connor and Davidson, 2003). Richardson (2002) resilience model points out that, as an individual’s intrinsically positive psychological resource, a high level of psychological resilience effectively promotes an individual’s positive personality traits so that he or she can effectively cope with the work and thus maintain a positive work engagement state. Meng et al. (2023) study also further pointed out that nurses with higher levels of psychological resilience have a strong sense of identity and belonging to their profession, which makes them willing to provide quality nursing care to patients, and this intrinsic motivation can further stimulate nurses’ work engagement (Meng et al., 2023). These studies have confirmed that psychological resilience has a positive effect on work engagement, but their mediating role is not fully explained.

Sense of occupational benefit refers to the fact that nurses can perceive the gains and benefits of nursing work and agree that the occupation they are engaged in can effectively promote self-growth (Hu et al., 2020). Studies have shown that nurses with a higher sense of occupational benefit are able to realize their self-worth from their work and develop their abilities, which motivates them to actively engage in their work (Zhuo and Zhang, 2022). Nurses with a high sense of career benefit have clear plans and goals for their career development, and they are able to continue to learn and improve their knowledge and skills, which can effectively enhance their commitment to their work (Zhou et al., 2019). In addition, according to the benefit discovery theory, individuals with high psychological resilience have positive career perceptions, and are able to effectively perceive the value and honor brought by their career, thus enhancing their work engagement (Zhao et al., 2021). Considering the above relationship between psychological resilience and work engagement, Hypothesis 1 is proposed: Perceived career benefits mediate the relationship between psychological resilience and work engagement.

Sense of career mission may be another influential factor between psychological resilience and work engagement. Sense of occupational mission refers to an individual’s love for the job he or she is doing, while perceiving the responsibility and mission given by the occupation (Zhang, 2022). When an individual has a high level of psychological resources within him/her, the more he/she is inclined to the pursuit of high level values such as sense of professional mission. Nurses with a high sense of professional mission are more responsible for the health and lives of patients, and they are aware of the importance of their work to patients, and this sense of responsibility and mission will directly promote nurses’ active engagement in their work (Chen et al., 2022). Thus, Hypothesis 2 is formulated: the sense of professional mission mediates the relationship between psychological resilience and work engagement. At the same time, nurses with a higher sense of occupational benefit have higher job satisfaction, they can get positive feedback and rewards from their work, feel the value and significance of their occupation, and perceive the meaning and mission brought by their occupation, so that they are willing to actively engage in their work (Zhang and He, 2023). This leads to Hypothesis 3: Sense of career benefit and sense of career mission play a chain mediating role between psychological resilience and work engagement.

In summary, positive psychological resources and nurses’ intrinsically positive career perceptions have received extensive attention in the field of nursing, but fewer reports have elaborated on the mechanisms of nurses’ psychological resilience, sense of career benefit, sense of career mission and work engagement. While analyzing the correlation between the four, this study explores the direct impact of psychological resilience on work engagement, as well as the mediating role played by the sense of occupational benefit and the sense of occupational mission, with the aim of facilitating the improvement of nurses’ work engagement, reducing the turnover rate of nurses, and enabling them to provide patients with more quality health services to provide a reference basis.

## 2 Materials and methods

### 2.1 Participants

In this study, a cross-sectional survey was conducted in August-October 2022, and 1,032 nurses from 10 general hospitals in Sichuan Province were selected as survey respondents by convenience sampling. Among them, there are 3 Grade IIIA hospitals, 3 Grade IIIB hospitals and 4 Grade II hospitals. Inclusion criteria: obtaining a license to practice as a nurse and working in clinical nursing for ≥1 year; informed consent and voluntary participation in this survey. Exclusion criteria: nurses on leave or in training.

The sample size was calculated by multiplying the number of variables by 5–10 and expanding the sample size by 20% to take into account attrition rates such as invalid questionnaires. Sample size = (10 + 25 + 33 + 10 + 9) × 5–10 x (1 + 20%) = 522–1044. The final sample analyzed in this study was 1032. Among them, gender: 75 males (7.3%), 957 females (92.7%); age: 20–58 (30.15 ± 7.67) years old; educational level: 356 specialists (34.5%), 676 undergraduates and above (65.5%); marital status: 599 married (58.0%), 395 unmarried (38.3%), 37 divorced (3.7%). The study was approved by the Ethics Committee (No. 2020-04-141).

### 2.2 Methodology

#### 2.2.1 Survey instruments

##### 2.2.1.1 General information questionnaire

The questionnaire was compiled by the research team members themselves after discussion based on the research objectives, and mainly included general demographic information and work characteristic information. General demographic data: gender, age, marital status and education; work characteristics data: hospital level, personal monthly income, title, department, number of weekly night shifts, and whether or not they have been engaged in front-line anti-epidemic work.

##### 2.2.1.2 Psychological resilience scale

The Chinese version of the CD-RISC Compiled by Connor and Davidson in the United States in 2003 and revised by Xiao Nan et al in China for Chinese, it has been shown to possess high reliability and validity in China, with a Cronbach’s α coefficient of 0.910 (Connor and Davidson, 2003; Yu and Zhang, 2007). Chen et al. (2020) applied the CD-RISC to nurses and the Cronbach’s α coefficient of the CD-RISC was 0.953. The CD-RISC consists of 25 entries for the dimensions of Resilience (13 entries), Strength (8 entries), and Optimism (4 entries), and is rated on a Likert four-point scale from “never” to “always.” The scale consisted of 25 items in 3 dimensions: toughness (13 items), strength (8 items), and optimism (4 items), and was rated on a 4-point Likert scale ranging from 0 to 4, from “never” to “always,” and the total score was 0 to 100. The total score was 0–100, and the higher the score, the better the psychological resilience. The reliability of this scale was good, with a Cronbach’s alpha coefficient of 0.925.

##### 2.2.1.3 Occupational benefits scale

The Occupational Sense of Gain Scale developed by Chinese scholars Hu and Liu (2013) was used, which has been shown to have high reliability and validity in China, with a Cronbach’s α coefficient of 0.958. Liang et al. (2020) applied it to nurses, with a Cronbach’s α coefficient of 0.896. The scale was composed of 33 items in 5 dimensions, namely, positive occupational perceptions (7 items), good nurse-patient relationship (6 items), sense of belonging to the team (6 items), recognition by friends and relatives (6 items), and self-growth (8 items), using a 5-point Likert scale, from “strongly disagree” to “strongly agree.” The scale consisted of 33 items in five dimensions: positive career perception (7 items), good patient-nurse relationship (6 items), team belonging (6 items), friends and family (6 items), and self-growth (8 items), and was rated on a 5-point Likert scale ranging from “Strongly disagree” to “Strongly agree,” and the total score was 33–165. The total score ranged from 33 to 165, and the higher the score, the stronger the sense of career benefit. The reliability of the scale was good, with a Cronbach’s α coefficient of 0.893.

##### 2.2.1.4 Sense of professional mission scale

Compiled by Zhang (2015), which was mainly used to measure the level of employees’ sense of professional mission and it has been shown to have high reliability and validity in China, with a Cronbach’s α coefficient of 0.890. Cheng et al. (2019) applied it to a group of nurses, and obtained a Cronbach’s α coefficient of 0.927. The scale consisted of three dimensions: orientation (4 items), initiative (3 items) and altruism (3 items), and 10 items were rated on a Likert 5-point scale, with scores ranging from 1 to 5, from “not at all conforming” to “completely conforming.” The scale consists of 3 dimensions: orientation (4 items), initiative (3 items), and altruism (3 items). A total of 10 items were scored on a 5-point Likert scale, ranging from 1 to 5, from “not at all in line” to “fully in line.” The total score was 10–50, and the higher the score, the stronger the sense of professional mission. The reliability of this scale was good, with a Cronbach’s α coefficient of 0.845.

##### 2.2.1.5 Work engagement scale

Developed by foreign scholars Schaufeli et al. (2006), and revised by Li et al. (2013) Fuye in China for Chinese, it has been shown to possess high reliability and validity in China. The Cronbach’s α coefficient of the scale was 0.930. Wang Q. et al. (2022) applied the scale to nurses’ group, and the Cronbach’s α coefficient of the scale was 0.930. The scale consisted of 9 entries of 3 dimensions: vitality (three entries), dedication (three entries), and concentration (three entries), and was rated on a Likert seven-point scale from “never” to “every day.” The scale consists of nine entries in 3 dimensions: vitality (three entries), dedication (three entries), and concentration (three entries), and is based on a seven-point Likert scale, with scores ranging from 0 to 6, from “never” to “every day,” respectively. The higher the score, the higher the level of work engagement. Based on the mean value of the scores of the entries, ≤2 is a low level, >2 and <4 is a medium level, and ≥4 is a high level. The reliability of the scale in this study was good, with a Cronbach’s α coefficient of 0.910.

#### 2.2.2 Survey methods

The study was conducted in the form of a Questionnaire Star (Wen Juan Xing, wjx.cn) on a web-based platform. Firstly, the copy of this questionnaire was edited by the members of the research team, including the title, introduction to the questionnaire, the purpose of the survey, significance, methodology and notes on filling in the questionnaire, and at the same time, the questionnaire was implanted in the questionnaire star and pre-surveys were conducted, and then the questionnaire was debugged according to the results to confirm the final version; secondly, the head of the research team contacted the person in charge of the relevant hospitals, explaining to him or her the purpose, content and significance of the survey, and in the case of obtaining their Under the condition of obtaining their consent, the link and QR code of the questionnaire were sent to the nursing workgroup of the department; finally, the nurses of each department filled in the questionnaire under the principle of voluntary and non-compulsory under the condition of informed consent. All questionnaire entries were mandatory, and each IP account had only 1 chance to answer. To ensure the confidentiality of the results, the results of this study are all digitally coded and can only be viewed by members of the research team. A total of 1115 questionnaires were collected, and questionnaires with the same option serial number and answer time <120 s or >1200 s were regarded as invalid questionnaires, and 83 questionnaires were excluded, resulting in a total of 1032 valid questionnaires recovered, with an effective recovery rate of 92.6%.

#### 2.2.3 Statistical methods

The results were exported directly from the questionnaire star and analyzed using SPSS 25.0 statistical software, with the four variables and dimensions approximately normally distributed, and the relationships between the variables were explored using descriptive analyses, *t*-tests, one-way ANOVA, Pearson’s correlation analyses, multivariate linear hierarchical regression analyses. According to the results of Harman one-way test for common method bias test, if the first factor explanation rate <40%, indicating that the data of this study does not have serious common method bias; take Hayes compiled SPSS macro program PROCESS for mediation effect analysis, Bootstrap method to repeat the sampling 5000 times to calculate the 95% CI, if none of the results contain 0, that is, the mediation effect is significant. Differences were considered statistically significant at *P* < 0.05.

## 3 Results

### 3.1 Psychological resilience, sense of occupational benefit, sense of occupational mission and work engagement scores of nurses

In this group 1032 nurses, with work engagement scores of (34.99 ± 9.80); psychological resilience scores of (91.29 ± 17.38); sense of occupational benefit scores of (137.85 ± 21.02); and sense of occupational mission scores of (40.27 ± 7.37) see Table 1 for details.

**TABLE 1:** Work engagement, psychological resilience, sense of professional benefit and sense of professional mission scores of nurses in this group (*n* = 1032).

Sports event	Entry	Scoring range	Score	Entry parity (accountancy)
Input	9	0–54	34.99 ± 9.80	3.89 ± 1.09
Vigor	3	0–18	11.58 ± 3.37	3.86 ± 1.12
Dedication	3	0–18	11.68 ± 3.62	3.89 ± 1.21
Single-mindedly devoted to	3	0–18	11.73 ± 3.44	3.91 ± 1.15
Psychological resilience	25	0–100	66.29 ± 17.38	5.10 ± 1.34
Tough and durable	13	0–52	33.90 ± 9.43	2.61 ± 0.73
Force	8	0–32	22.12 ± 5.58	2.77 ± 0.70
Pessimistic	4	0–16	10.27 ± 3.01	2.57 ± 0.75
Sense of career benefits	33	33–165	137.85 ± 21.02	4.18 ± 0.64
Positive career perception	7	7–35	28.68 ± 5.01	4.10 ± 0.72
Nurse-patient relationship	6	6–30	25.02 ± 4.06	4.17 ± 0.68
Recognition by family and friends	6	6–30	24.64 ± 4.23	4.11 ± 0.70
Team affiliation	6	6–30	25.76 ± 4.02	4.29 ± 0.67
Self-growth	8	8–40	33.75 ± 5.27	4.22 ± 0.66
Professionalism	10	10–50	40.27 ± 7.37	4.03 ± 0.74
Conductivity	4	4–20	15.46 ± 3.41	3.86 ± 0.85
Altruistic force	3	3–15	12.57 ± 2.23	4.19 ± 0.74
Proactive and aggressive	3	3–15	12.24 ± 2.33	4.03 ± 0.74

### 3.2 Comparison of work engagement scores of nurses with different characteristics

The results of univariate analysis showed that the differences in the total work engagement scores of nurses with different genders, hospital grades, ages, education levels, marital statuses, personal monthly incomes, and titles were statistically significant (all *P* < 0.05), as shown in Table 2.

**TABLE 2:** Comparison of work engagement scores of nurses with different characteristics (*n* = 1032).

Sports event		Number of persons (per cent)	Score x ± s	*F/t*	*P*
Distinguishing between the sexes	Male	75 (7.3)	33.56 ± 7.83	−1.608	0.111
	Women	957 (92.7)	35.10 ± 9.93		
Hospital level	Grade 3A	482 (46.7)	36.98 ± 9.46	37.046	<0.001
	Grade 3B	314 (30.4)	35.30 ± 9.31		
	Level II hospitals	236 (22.9)	34.99 ± 9.80		
Age (years)	20 to	404 (39.1)	35.78 ± 9.04	3.650	0.0123
	30 to	512 (49.6)	37.65 ± 9.57		
	40 to	96 (9.3)	37.69 ± 7.68		
	50 to	20 (2.0)	38.85 ± 9.84		
Academic qualifications	Branch (of medicine)	356 (34.5)	32.20 ± 9.86	−6.787	<0.001
	Undergraduate and above	676 (65.5)	36.46 ± 9.44		
Marital status	Married	599 (58.0)	35.42 ± 9.77	3.774	0.023
	Unmarried	395 (38.3)	34.07 ± 9.98		
	Divorcee	37 (3.7)	37.70 ± 7.46		
Monthly personal income ($)	0 ∼	466 (45.2)	33.46 ± 9.84	17.530	<0.001
	5000 ∼	458 (44.4)	35.53 ± 9.60		
	10001 ∼	108 (10.4)	39.33 ± 8.94		
Title	Junior ranking	613 (59.4)	34.04 ± 9.97	7.375	0.001
	Middle level (in a hierarchy)	353 (34.2)	36.26 ± 9.31		
	High level	66 (6.4)	37.05 ± 9.80		
Sections	General medicine	476 (46.1)	35.39 ± 10.08	1.018	0.362
	Neurosurgery	363 (35.2)	34.42 ± 10.00		
	Acute and critical illness	193 (18.7)	35.09 ± 8.62		
Number of weekly night shifts (times)	0	313 (30.3)	35.52 ± 9.59	2.580	0.052
	1	173 (16.8)	36.39 ± 9.68		
	2	366 (35.5)	34.30 ± 9.61		
	≥3	180 (17.4)	34.12 ± 10.49		
Whether or not they have worked in the fight against epidemics	Be	453 (43.9)	35.55 ± 10.16	1.595	0.111
	Clogged	579 (56.1)	34.56 ± 9.49		

### 3.3 Correlation of psychological resilience, sense of occupational benefit, sense of occupational mission and work engagement of the nurses

In this group *Pearson* correlation analysis showed that the total score of psychological resilience of the nurses in this group was positively correlated with the total scores of sense of occupational benefit, sense of occupational mission and work engagement (*r* = 0.625–0.806, both *P* < 0.01); the total scores of sense of occupational benefit were positively correlated with sense of occupational mission and work engagement (*r* = 0.705–0.842); and sense of occupational mission was positively correlated with work engagement (*r* = 0.725, both *P* < 0.01). correlation (*r* = 0.705 to 0.842, both *P* < 0.01); the sense of career mission and the total score of work engagement were positively correlated (*r* = 0.725, both *P* < 0.01) see Table 3.

**TABLE 3:** Correlation analysis of psychological resilience, sense of professional gain, sense of professional mission, and work engagement among nurses in this group (*n* = 1032, *r*).

Sports event	Input	Vigor	Dedica-tion	Single-mindedly devoted to	Psycho-logical resili-ence	Tough and durable	Force	Pessi-mistic	Sense of career benefits	Positive career percep-tion	Nurse-patient rela-tionship	Recogni-tion by family and friends	Team affilia-tion	Self-growth	Profes-siona-lism	Conduc-tivity	Altruis-tic force	Pro-active and aggres-sive
Input	1.000																	
Vigor	0.940**	1.000																
Dedication	0.944**	0.837**	1.000															
Single-mindedly devoted to	0.934**	0.816**	0.817**	1.000														
Psychological resilience	0.806**	0.785**	0.764**	0.721**	1.000													
Tough and durable	0.779**	0.759**	0.737**	0.699**	0.983**	1.000												
Force	0.802**	0.774**	0.766**	0.718**	0.965**	0.913**	1.000											
Pessimistic	0.727**	0.722**	0.683**	0.643**	0.909**	0.850**	0.857**	1.000										
Sense of career benefits	0.705**	0.682**	0.680**	0.625**	0.736**	0.708**	0.753**	0.638**	1.000									
Positive career perception	0.702**	0.680**	0.693**	0.605**	0.714**	0.688**	0.723**	0.630**	0.940**	1.000								
Nurse-patient relationship	0.598**	0.569**	0.579**	0.536**	0.593**	0.553**	0.652**	0.484**	0.873**	0.807**	1.000							
Recognition by family and friends	0.639**	0.628**	0.603**	0.569**	0.700**	0.686**	0.690**	0.615**	0.920**	0.820**	0.692**	1.000						
Team affiliation	0.668**	0.652**	0.640**	0.591**	0.716**	0.689**	0.724**	0.634**	0.950**	0.873**	0.784**	0.864**	1.000					
Self-growth	0.663**	0.634**	0.637**	0.597**	0.693**	0.668**	0.709**	0.596**	0.960**	0.855**	0.793**	0.895**	0.900**	1.000				
Professiona-lism	0.725**	0.686**	0.702**	0.653**	0.733**	0.712**	0.732**	0.646**	0.842**	0.823**	0.676**	0.775**	0.790**	0.828**	1.000			
Conductivity	0.714**	0.690**	0.685**	0.637**	0.708**	0.700**	0.683**	0.630**	0.740**	0.731**	0.529**	0.718**	0.705**	0.736**	0.926**	1.000		
Altruistic force	0.600**	0.553**	0.577**	0.559**	0.627**	0.597**	0.647**	0.549**	0.788**	0.765**	0.707**	0.689**	0.723**	0.768**	0.893**	0.684**	1.000	
Proactive and aggressive	0.673**	0.631**	0.665**	0.599**	0.683**	0.657**	0.697**	0.597**	0.824**	0.803**	0.688**	0.742**	0.775**	0.808**	0.953**	0.810**	0.866**	1.000

**P < 0.001.

### 3.4 The chain mediating role of sense of professional gain and sense of professional mission between nurses’ psychological resilience and work engagement

#### 3.4.1 Common method bias test

According to the results of Harman’s one-way test, there are seven factors with eigenroots >1 and the first factor has an explanatory rate of 32.630%, which is less than 40%. It indicates that there is no serious common method bias in the data of this study.

#### 3.4.2 Tests of mediating effects of variables

**FIGURE 1:**
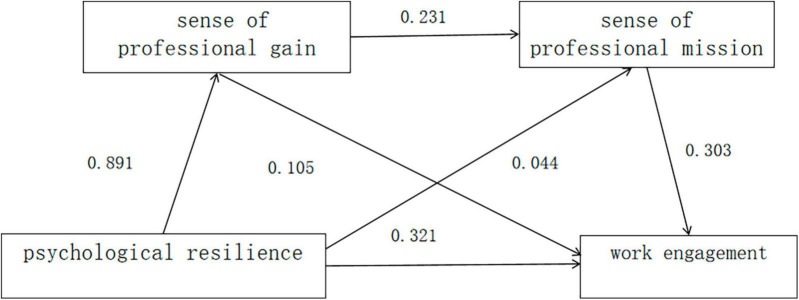
Chain mediation model of career sense of gain and career sense of purpose between nurses’ psychological resilience and work engagement (*n* = 1032).

The results showed that psychological resilience positively predicted sense of career benefit (*P* < 0.05), psychological resilience and sense of career benefit positively predicted sense of career vocation (both *P* < 0.05), and psychological resilience, sense of career benefit, and sense of career vocation positively predicted work engagement (all *P* < 0.05) see Table 4 and Figure 1.

**TABLE 4:** Regression models for the chain mediation model of professional sense of gain and professional sense of purpose in psychological resilience and work engagement of nurses (*n* = 1032).

Regression equation		Fitness index			Significance of regression coefficients	
**Outcome variable**	**Predictor variable**	***R***	***R* ^2^**	***F***	**β**	***T***
Sense of career benefits	Psychological resilience	0.736	0.542	1220.379	0.891	34.933**
Professionalism	Psychological resilience	0.858	0.690	1438.549	0.105	10.483**
	Sense of career benefits				0.231	27.869**
Input	Psychological resilience	0.831	0.737	769.914	0.321	21.079**
	Sense of career benefits				0.044	2.776**
	Professionalism				0.303	6.749**

**P < 0.01.

The results of the mediation effect analysis showed that the 95% CI for all paths did not contain 0, and the mediation effect was established. The results of mediated effect analysis showed that the indirect effect of psychological flexibility on work engagement can be through three paths: ① psychological flexibility → sense of career benefit → work engagement; ② psychological flexibility → sense of career mission → work engagement; ③ psychological flexibility → sense of career benefit → sense of career mission → work engagement. Among them, the total indirect effects accounted for 29.39%, including 8.62% of the mediating path ①, 7.02% of the mediating path ② and 13.75% of the mediating path ③ see Table 5.

**TABLE 5:** Analysis of the mediating effects of sense of professional gain and sense of professional mission in nurses’ psychological resilience and work engagement (*n* = 1032).

Trails	Path effects of standardization	Amount of effect/per cent	95 percent *CI*	
			**Lower limit**	**Limit**
Psychological resilience–sense of professional gain–work engagement	0.891 × 0.044 = 0.0392	8.62	0.0099	0.0705
Psychological Resilience–Sense of Professional Mission–Work Engagement	0.105 × 0.303 = 0.0319	7.02	0.0225	0.0435
Psychological resilience–Sense of professional gain–Sense of professional mission–Work engagement	0.891 × 0.231 × 0.303 = 0.0624	13.75	0.0428	0.0823
indirect effect	0.0392 + 0.0319 + 0.0624 = 0.1335	29.39	0.1091	0.1572
Direct effect	0.3207	70.61	0.2908	0.3505
Aggregate effect	0.4542	100.00	0.4338	0.4746

## 4 Discussion

### 4.1 Nurses in this group are at the middle level in terms of work engagement and psychological resilience, and their sense of occupational benefit and sense of occupational mission are at the middle to upper level

The average value of the entry for nurses’ work engagement in this group is (3.89 ± 1.09), which is at the middle level compared with the middle value of the entry assigned to 3, and is higher than that of the survey conducted by Yang et al. (2022) on a tertiary care hospital. The reasons for this are: (1) 43.9% of the nurses in this group have participated in the front line prevention and control of epidemics, and the study found that the nurses who have participated in the front line prevention and control of epidemics understand the importance of their own work, and are able to show a high degree of responsibility and concentration in their clinical work, as well as being willing to devote themselves to their work with enthusiasm (Yan et al., 2021); (2) 18.7% of the nurses in this group come from the acute and critical care units, and the patients’ conditions that they face are mostly more critical, making it necessary for nurses to work in the hospitals of the three major hospitals. Most of the nurses are in critical condition, which makes the nurses must have a high degree of dedication to focus on their own clinical work, and thus have a relatively high degree of commitment to their work; and (3) with the steady development of the nursing career, the implementation of the scientific and feasible performance reform programme and the salary system, the professional development of the nurses and their income have been correspondingly improved, which has a contributing effect on the nurses’ motivation to work from the side of the (Yang et al., 2021).

The psychological resilience score of the nurses in this group is at a medium level, higher than the study of Li et al. (2020), but lower than the foreign related studies (Walpita and Arambepola, 2022). The reason for this is that 60.9% of the nurses in this group were >30 years old, compared with longer working years, experienced the impact of the transition of their careers, had relatively stable jobs, and possessed a certain degree of self-responsiveness to deal with the dilemmas faced by their clinical work; however, the dilemmas of the shortage of nursing human resources continue to exist in China at the present time, and the nurses’ workloads are heavy and stressful, which in turn affects the improvement of the level of psychological resilience.

The scores of nurses’ sense of occupational benefit in this group were at a medium-high level, which was basically consistent with the results of the study conducted by Fang et al. (2021). The reason for this is that with the continuous advancement of healthcare reform, the clinical working environment and treatment of nurses have been further improved, leading to the enhancement of their sense of identification with their profession; at the same time, with the advancement of Internet + nursing services, more and more nurses can take orders online for offline nursing services, and the professional value of nurses has been further enhanced, resulting in a significantly stronger perception of benefits to the profession (Zhang et al., 2022).

The scores of nurses’ sense of professional mission in this group are at a medium-high level, which is consistent with the results of the study by Li et al. (2021) and higher than the study of teachers by Xie and Chen (2021). The reason for this is that due to the special nature of the profession, nurses undertake the important task of saving lives and helping the injured, and their professionalism requires a high degree of social responsibility and sense of professional mission, which results in relatively high scores; at the same time, 43.9% of the nurses in this group have been involved in the first-line prevention and control of epidemics, and since the outbreak of the epidemic, nurses, as the main force of the epidemic prevention, have fully embodied their personal value, which has enabled them to understand that the work they are engaged in requires them to shoulder the responsibility of helping others and contributing to the health of the community. The nurses’ personal value as the main force in epidemic prevention has been fully embodied, making them understand that their work requires them to shoulder the responsibility of helping others and contributing to the health of the society accordingly, thus showing a great sense of pride and satisfaction, and further promoting the enhancement of the sense of professional mission (Wang et al., 2020).

### 4.2 The total psychological resilience scores of the nurses in this group were positively correlated with the total scores of career benefits, career mission and work commitment, and the total scores of career benefits were positively correlated with the total scores of career mission and work commitment, and the total scores of career mission and work engagement were positively correlated with the total scores of work engagement

The results of this study showed that the psychological resilience of the nurses in this group was positively correlated with the sense of career gain (*r* = 0.625, *P* < 0.001), i.e., the higher the psychological resilience of the nurses, the stronger their sense of career gain, which is consistent with the results of the study (Sun, 2022). The reason for this is that when nurses have a high level of psychological resilience, they are able to positively challenge difficult work situations and at the same time feel proud of their achievements in overcoming difficulties, thus increasing their sense of career gain. The results of this study showed that the psychological resilience of this group of nurses was positively correlated with the sense of professional mission (*r* = 0.733, *P* < 0.001), i.e., the higher the psychological resilience of the nurses, the stronger their sense of professional mission, which is consistent with the results of the study (Xu et al., 2020). The reason for this is that the more psychologically resilient individuals have a high degree of strength and conviction to shoulder the mission and responsibility given by their profession. The results of this study showed that the psychological resilience of this group of nurses was positively correlated with work engagement (*r* = 0.806, *P* < 0.001), i.e., the higher the psychological resilience of the nurses, the more they were willing to engage in their work, which is consistent with the findings of Yu (2022). The reason for this is that psychological elasticity as an internal driving force can effectively promote the nurses to actively engage in their work.

The results of this study showed that there was a positive correlation between the nurses’ sense of occupational benefit and their sense of professional mission in this group (*r* = 0.705, *P* < 0.001), which means that the higher the nurses’ sense of occupational benefit, the stronger their sense of professional mission. It may be that nurses with a higher sense of occupational benefit are good at communicating with patients and their families, and are able to obtain their recognition and approval, which further enhances occupational value and satisfaction. The results of this study showed that the nurses’ sense of occupational benefit was positively correlated with work engagement in this group (*r* = 0.842, *P* < 0.001), i.e., the higher the nurses’ sense of occupational benefit, the more they were engaged in their work, which is consistent with the findings of Wang et al. (2017). The reason for this is that when nurses perceive that nursing can enable them to give full play to their own strengths and realize their own social value, they are bound to be full of confidence and vitality, and will be actively involved in it.

The results of this study showed a positive correlation between nurses’ sense of professional mission and work engagement in this group (*r* = 0.725, *P* < 0.001), i.e., the higher the nurses’ sense of professional mission is, the more they are committed to their work, which is consistent with the findings of Yu and Liu (2020). The possible reason for this is that when nurses believe that the work they do is beneficial to others and meets the needs and development of society, they will give their time and energy for their profession.

### 4.3 Analysis of chained mediation effects between variables

#### 4.3.1 The mediating role of occupational profitability in the relationship between nurses’ psychological resilience and work engagement

The results of this study showed that nurses’ psychological resilience positively predicted the sense of occupational benefit (β = 0.891, *P* < 0.001), while the sense of occupational benefit also positively influenced work engagement (β = 0.044, *P* < 0.001). After the mediation effect test of Bootstrap method, there was a mediation effect between psychological resilience and work engagement, and the mediation effect value was 0.039. Psychological resilience, as an important part of positive psychology, helps nurses to adapt to stressful changes in stressful environments, to actively explore the positive parts of work, and to further perceive the benefits and values of clinical work for their own development (Clark et al., 2021). At the same time, nurses with a high level of occupational benefit have positive perceptions of their occupation, which can be effectively transformed into emotional identification with nursing work, which can further stimulate their intrinsic motivation and make them spontaneously devote themselves to their work with positive enthusiasm and full energy. It is suggested that managers should pay attention to the cultivation of nurses’ psychological resilience and strengthen the enhancement of professional value and sense of benefit, such as carrying out training on positive coping and psychological construction under stressful events, creating a magnetic nursing environment, strengthening humanistic care support, enhancing professional value, and providing nurses with effective organizational and social resources, so as to enable them to obtain a sense of occupational satisfaction and pride in their nursing work, in order to further enhance the behavior of work engagement, to Reduce willingness to leave and maintain nursing team stability (Pan et al., 2019).

#### 4.3.2 The mediating role of professional mission between nurses’ psychological resilience and work engagement

The results of this study showed that nurses’ psychological resilience positively predicted the sense of professional mission (β = 0.105, *P* < 0.001), and the sense of professional mission also positively affected work engagement (β = 0.303, *P* < 0.001). After the mediation effect test of Bootstrap method, the sense of professional mission had a mediation effect between psychological resilience and work commitment, and the value of mediation effect was 0.032. Psychological resilience is one of the important components of psychological capital, and nurses with strong psychological resilience have positive behaviors toward their own work, can effectively cope with work pressure, and are good at career exploration, so that they have more chances to sense the sense of achievement and responsibility and mission given to them by the profession. They have more opportunities to perceive the sense of achievement and responsibility that comes with their profession. According to the Nahrgang et al. (2011) theoretical model, the sense of professional mission, as a strong internal driving force, can fully stimulate nurses’ potential and passion for work, improve their recognition of their work, and thus make them more focused and committed to their work. This suggests that managers should pay attention to the impact of nurses’ psychological flexibility on the sense of professional mission, which can start from increasing work-based support resources, and through the enhancement of psychological flexibility to further bring into play the intrinsic leverage of the sense of professional mission, so that nurses can focus on their own work, and be more proactive in completing the established work goals.

#### 4.3.3 Chain mediating role of sense of professional gain and sense of professional mission between nurses’ psychological resilience and work engagement

The results of this study showed that nurses’ psychological resilience positively predicted their sense of career benefit (β = 0.891, *P* < 0.001), their sense of career benefit positively predicted their sense of career mission (β = 0.231, *P* < 0.001), and their sense of career mission positively affected their work engagement (β = 0.303, *P* < 0.001). The mediating effect of the Bootstrap method showed that there was a chain mediating effect between psychological resilience and work engagement, with a chain mediating effect value of 0.062. Nurses with strong psychological resilience were able to cope with difficult situations in nursing, had a high degree of professional decision-making and development planning, and showed professional belongingness to the work they were engaged in, which increased their level of professional benefit (He et al., 2019). As an internal emotional experience and endogenous motivational factors, when their own values are satisfied, they can further mobilize their internal motivation for work, fully stimulate their subjective initiative, and produce positive professional emotional experience, further strengthen the nurses’ perception of their work, make them understand the value and significance of the work they are engaged in, and then be willing to shoulder the corresponding responsibilities, thus promoting the nurses to have high levels of Sense of professional mission. Sense of professional mission as a professional forward momentum, will prompt nurses as a medical worker to save lives and help the injured feel proud, so that it more recognition and acceptance of the current nursing work, and can be organizational development and the health of the patient as a sense of responsibility for the knowledge, and more actively involved in the work. Tips for managers: (1) should be reasonable human resource allocation, reduce the work pressure of nurses, improve their psychological resilience level; (2) build organizational support system, create a humane management atmosphere, appropriate authorization to allow nurses to participate in the daily work management, in order to play a positive dynamic role for the organizational development of the advice, so as to enhance their sense of professional achievement; (3) to strengthen the positive publicity of the work of nursing, so that doctors, and (4) strengthen the positive publicity of nursing work, so that doctors, patients and their families can have a clear perception of nursing work and give positive evaluation, so that nurses feel that their own efforts can be recognized and supported accordingly, in order to further awaken their professional mission, so that they will be more willing to focus on their own clinical work and provide patients with high-quality nursing services.

## 5 Limitation

There are still some limitations of this study. Firstly, this study took the form of online questionnaire to fill in, and the results of the study are a little subjective. Secondly, this study only chose 10 general hospitals in Sichuan Province for the survey, and the sample is not representative enough. Finally, this study only conducted a cross-sectional survey, no longitudinal study was conducted to view the change trend of the scale, and also no corresponding intervention study was conducted. In future studies, we can adopt multi-region and multi-sample studies, as well as longitudinal dynamic observation, in order to provide a reference basis for the subsequent development of interventions to improve nurses’ work engagement.

## 6 Conclusion

Nurses’ work engagement is at a medium level, and the chain mediating role of occupational benefit and sense of mission in the mechanism of nurses’ psychological resilience on work engagement is established. This suggests that managers should pay attention to nurses with low psychological elasticity, provide them with appropriate organizational support and create a harmonious organizational atmosphere and environment through appropriate load-shedding and motivational mechanisms, so as to enhance the acquisition of their sense of occupational benefit and sense of mission, and to make them more committed to their work.

## Data availability statement

The raw data supporting the conclusions of this article will be made available by the authors, without undue reservation.

## Ethics statement

The studies involving humans were approved by the Medical Ethics Committee of Deyang People’s Hospital. The studies were conducted in accordance with the local legislation and institutional requirements. The participants provided their written informed consent to participate in this study.

## Author contributions

ZL: Writing – original draft, Writing – review and editing, Data curation, Investigation, Supervision. CC: Writing – review and editing. XY: Investigation, Writing – original draft. JW: Investigation, Writing – review and editing. LL: Data curation, Supervision, Writing – review and editing.
